# PDIA3 Knockdown Exacerbates Free Fatty Acid-Induced Hepatocyte Steatosis and Apoptosis

**DOI:** 10.1371/journal.pone.0133882

**Published:** 2015-07-27

**Authors:** Xue-qun Zhang, Yue Pan, Chao-hui Yu, Cheng-fu Xu, Lei Xu, You-ming Li, Wei-xing Chen

**Affiliations:** 1 Department of Gastroenterology, the First Affiliated Hospital, College of Medicine, Zhejiang University, Hang Zhou, Zhejiang Province, China; 2 Department of Gastroenterology, Ningbo No.1 Hospital, Ningbo, Zhejiang Province, China; Bambino Gesu' Children Hospital, ITALY

## Abstract

Nonalcoholic fatty liver disease (NAFLD) has emerged as one of the most common chronic liver disease over the past decades. Endoplasmic reticulum stress (ERS) plays a pivotal role during the development of NAFLD. This study aims to analyze the potential role of protein disulfide isomerase A3 precursor (PDIA3), one of the ER chaperones, in free fatty acid-induced cell model of NAFLD. Human liver L02 cell line was treated with sodium palmitate for 24 hours, which developed severe intracellular lipid accumulation. The increased protein level of PDIA3 was detected via immunoblotting analysis in the fat loaded cell models of NAFLD. siRNA-mediated knockdown of PDIA3 in L02 cells not only increased the cellular lipid accumulation, but also exacerbated hepatocytes apoptosis induced by sodium palmitate. Further investigation revealed that knockdown of PDIA3 up-regulated protein expression of fatty acid synthase (FAS), a key enzyme involved in fatty acid synthesis. PDIA3 knockdown also up-regulated key molecules of ERS pathway, including glucose-regulated protein 78 (GRP78), phospho-PKR-like ER kinase (p-PERK), and C/EBP homologous protein (CHOP). Our results suggested that ER chaperone PDIA3 plays a pivotal role in FFA-induced hepatocyte steatosis and apoptosis.

## Introduction

Nonalcoholic fatty liver disease (NAFLD) is a chronic metabolic syndrome characterized by fat accumulation in the liver, with the exception of alcohol and other known causes of liver damage. The prevalence of NAFLD in Western countries has reached 20–30%, meanwhile, the high prevalence has been observed in Eastern countries [1,2]. NAFLD includes a series of interconnected clinical pathological syndromes, ranging from steatosis to nonalcoholic steatohepatitis (NASH), which can progress to fibrosis, cirrhosis, and eventually to liver failure or hepatocellular carcinoma [3,4]. The underlying mechanism of NAFLD has not been fully elucidated. The “two-hit” hypothesis is currently the most recognized theory to explain disease development and progression [5]. It points out that the development of simple steatosis is the first hit, on the basis, hepatocytes are more sensitive to damage factors such as mitochondrial dysfunction, oxidative stress, adipocytokine alteration and lipid peroxidation, which finally leads from simple steatosis to NASH.

Recently, growing evidences have shown that endoplasmic reticulum stress (ERS) plays a pivotal role in both the development of steatosis and progression to NASH [6,7]. A variety of factors such as anoxia, oxidative stress, alcohol, drugs and virus infection break the homeostasis of endoplasmic reticulum and cause unfolded and/or misfolded proteins accumulation in the endoplasmic reticulum lumen, which induce a physiological reaction referred to as the unfolded protein response (UPR) [8–11]. The UPR is mediated by three different signaling pathways, each of which is initiated by a specific transmembrane protein anchored in the endoplasmic reticulum membrane: protein kinase dependent on RNA (PKR)-like ER kinase (PERK), inositol-requiring enzyme 1 (IRE1), and activating transcription factor 6 (ATF6). The UPR is primarily a cytoprotective survival response that aims to regulate protein folding and restore homeostatic balance. However, chronic or unresolved ERS can lead to apoptosis [8]. A large sum of researches have indicated that ERS may lead to activation of various signal pathways that has been linked to insulin resistance, inflammation, and apoptotic cell death, all of which are important in the pathogenesis of NAFLD [12–14].

Elevated circulating free fatty acids (FFA) are a character of NAFLD, however, increased FFA, especially long-chain saturated fatty acids (such as palmitic acid), can induce an ERS response that can activate stress signaling pathways, cause hepatocyte cell death and liver damage [15,16]. The mechanisms by which saturated fatty acids contribute to liver injury remain poorly understood.

Protein disulfide isomerase A3 precursor (PDIA3) is a component of the protein disulfide isomerase family and distributes mainly in the endoplasmic reticulum lumen [17]. PDIA3 functions as parts of the glycoprotein-specific quality control machinery operating in the lumen of the ER [17]. When ERS is activated, the expression levels of PDIA3 and other ER chaperones are induced to increase the folding capacity of the ER. It was reported that PDIA3 was highly induced in neurodegenerative disease like prion disease and its overexpression protects neuronal cells against PrP^sc^ toxicity and ERS-induced apoptosis [18,19]. Our previous study also reported that expression of PDIA3 was up-regulated in livers of rats fed high-fat diet [20]. However, the specific molecular mechanism of PDIA3 in NAFLD remains poorly understood. We assumed that PDIA3 participate in the pathogenesis of NAFLD through ERS, and the present study aimed to explore the underlying association in vitro.

## Materials and Methods

### Cell culture

Normal human hepatocytes L02 cells were obtained from China Cell Culture Center (Shanghai). The cells were cultured in Dulbecco's Modified Eagle Medium (DMED; Gibco, Carlsbad, CA) supplemented with 10% fetal bovine serum (FBS; Gibco) and incubated at 37°C in a humidified atmosphere of 5% CO_2_. In order to establish fat overloading cell models, FFA (sodium palmitate (Sigma, St.Louis., MO) was added at a final concentration of 0.5 mM for 24 hours.

### Small interfering RNA (siRNA) transfection

For RNA silencing, four different sequences of siRNA targeting human PDIA3 were designed and synthesized by GenePharma (Shanghai, China) as follows: siRNA1: 5’-CCAGCAACUUGAGGGAUAATTUUAUCCCUCAAGUUGCUGGTT-3’;

siRNA2: 5’-GGACAAGACUGUGGCAUAUTTAUAUGCCACAGUCUUGUCC

TT-3’; siRNA3: 5’-CAGCCAACAAGAAGCUAAATTUUUAGCUUCUUGUUG

GCUGTT-3’; siRNA4: 5’-GGACGGUCAUUGAUUACAATTUUGUAAUCAA

UGACCGUCCTT-3’. Nonsilencing siRNA was a commercially available duplex (GenePharma) that was used as a negative control siRNA. For gene silencing, L02 cells were transfected with 160pmol siRNA using the Lipofectamine 2000 (Invitrogen, Carlsbad, CA) as instructed by the manufacturer. Forty-eight hours after transfection, protein extracts were analyzed by Western Blot to confirm protein knockdown. To assess the influence of PDIA3 knockdown on cellular steatosis and apoptosis, cells were treated with FFA after 48 hours of siRNA transfection and harvested after 24 hours incubation with FFA.

### Oil Red O Staining

Cells were washed twice with PBS before fixed with 10% formaldehyde in PBS for 15 minutes. After two washes in PBS, cells were then stained for 15 minutes in freshly diluted Oil Red O solution. After that, the dishes were rinsed in water and counterstained with hematoxylin for 1 minute. Representative micrographs were captured at 200× magnification using a microscope.

### Triglyceride assay

Intracellular triglycerides were assayed using a triglyceride assay kit (GPO-POD; Applygen Technologies Inc., Beijing, China) according to the manufacturer’s recommended protocol.

### Apoptosis analysis

Early and late phase apoptotic cells were detected with AnnexinV-FITC Apoptosis assay (KeyGen, Nanjing, China). Cells were washed twice with cold PBS, resuspended in binding buffer, and then incubated with Annexin V-fluorescein isothiocyanate (FITC) and propidium iodide (PI) staining solution following the manufacturer’s instructions. Samples of 10000 stained cells were analyzed using a FACSCalibur flow cytometer (BD Biosciences, San Jose, CA).

### Western Blot

Total protein was collected from cells using a Total Protein Extraction Kit (Sangon Biotech, Shanghai, China). Equal amounts of protein per sample were separated by 10% or 12% SDS-PAGE and transferred to polyvinylidene fluoride (PVDF) membranes. The membranes were blocked in 5% non-fat milk for 1 hour at 37°C and then incubated at 4°C overnight with primary antibodies, followed by incubation with horseradish peroxidase-conjugated secondary antibodies at 37°C for 1 hour. Primary antibodies against PDIA3, ATF-6α, p-PERK (Santa Cruz, CA), GRP78, CHOP, PERK, IRE1, SREBP1, FAS, ACC1 (Cell Signaling Technology, Danvers, MA) and p-IRE1 (Abcam, UK) were selected for this study. GAPDH (Cell Signaling Technology) was used as a control. The secondary antibodies were purchased from Santa Cruz Biotechnology. The immunoreactive bands were detected with ECL reagents and exposed to x-ray film for visualization. The density of each protein band was quantified by normalization to GAPDH.

### Statistical analysis

Data were given as mean ± SD from at least three independent experiments. Independent samples t–tests were done between two groups of continuous variables. *P* values <0.05 were regarded as statistically significant. All analyses were performed with Graphpad Prism 5.01 software.

## Results

### The expression level of PDIA3 was increased in fat loaded cell models of NAFLD

**Fig 1:**
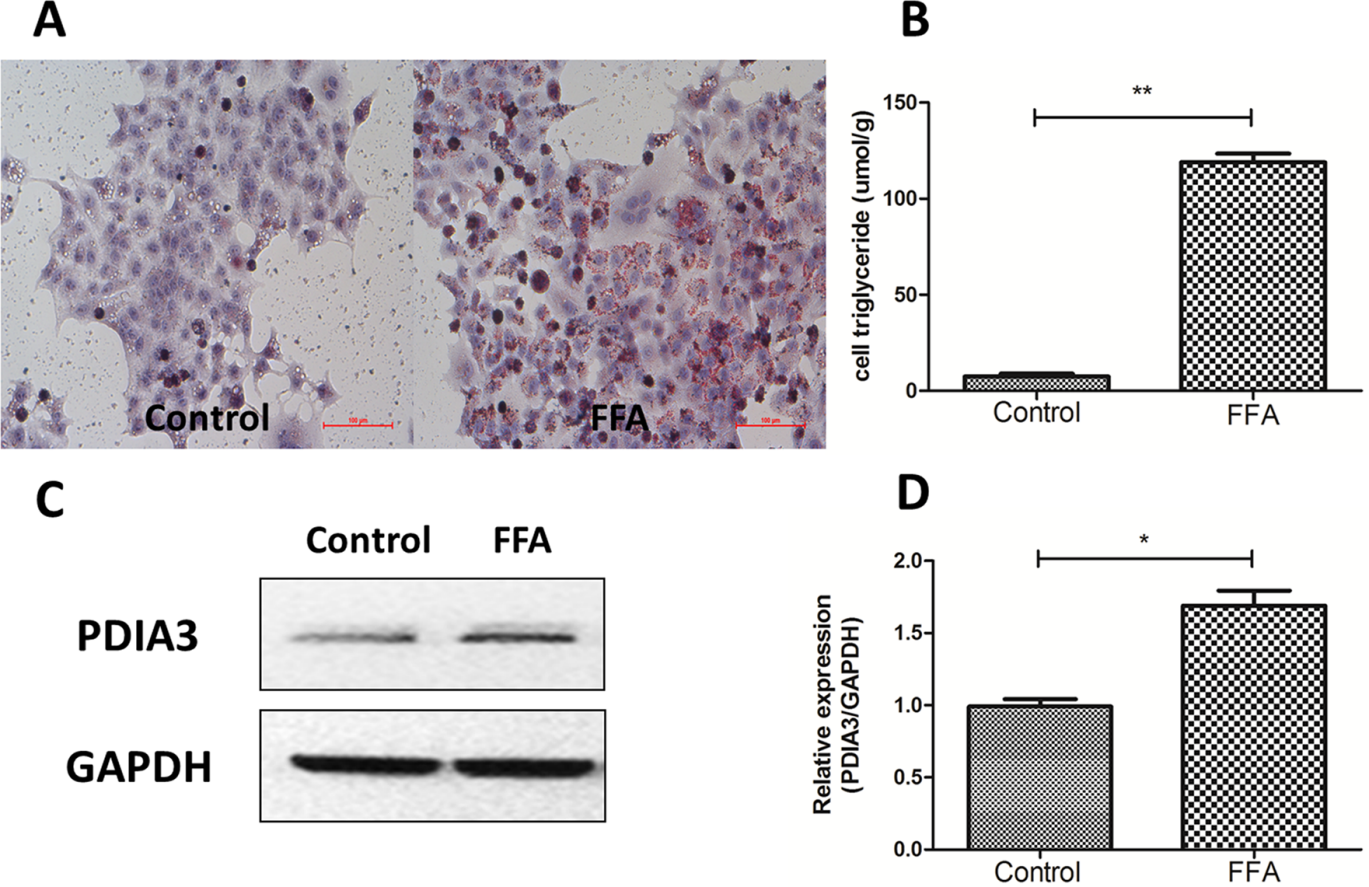
The expression level of PDIA3 was increased in fat loaded cell models of NAFLD. (A) Oil red O staining. Original magnification, 200×. (B) Intracellular triglyceride assay. **P<0.01. (C) Western blot analysis of PDIA3 protein expression. (D) The relative expression level quantified by densitometry analysis of bands and normalized to GAPDH protein. All studies were conducted at least three times in triplicate, *P<0.05.

In order to establish fat overloading cell models, the human hepatocyte cell line L02 cells were treated with 0.5mM sodium palmitate for 24 hours. Oil red O staining demonstrated that lipid droplets were increased when L02 cells were treated with FFA for 24 hours (Fig 1A), and the triglyceride assay showed the accordingly results (Fig 1B; *P*<0.01). On this basis, we found that the expression level of PDIA3 was increased in fat loaded cell models (Fig 1C and 1D; *P*<0.05). The results were in accordance with our previous study in high fat diet-induced rat models of NAFLD [20].

### PDIA3 siRNA interference effect

**Fig 2:**
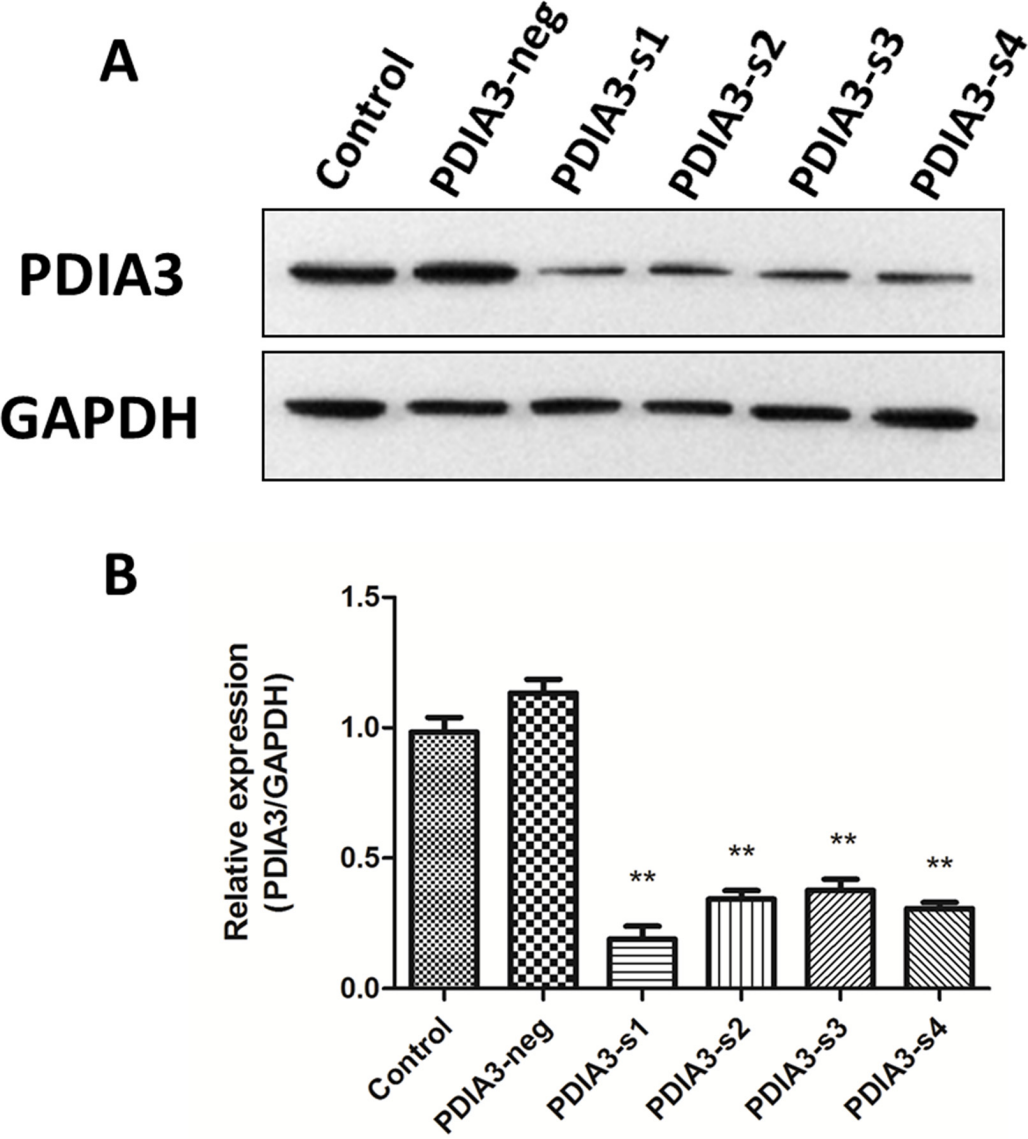
PDIA3 siRNA interference effect. (A) PDIA3 siRNA markedly reduces the PDIA3 protein level in L02 cells. (B) Relative protein level of PDIA3 after siRNA transfection. The PDIA3-s1 has the most significant knockdown effect. **P<0.01 versus negative siRNA transfected cells. Three independent experiments were performed.

Four pairs of siRNA duplexes were designed and synthesized for down-regulation of PDIA3 in L02 cells. Western Blot showed that the knockdown effect of the first sequence was more significant than others and it was therefore used in the following experiments (Fig 2A and 2B; *P*<0.01).

### PDIA3 siRNA exacerbated hepatocellular steatosis in L02 cells

**Fig 3:**
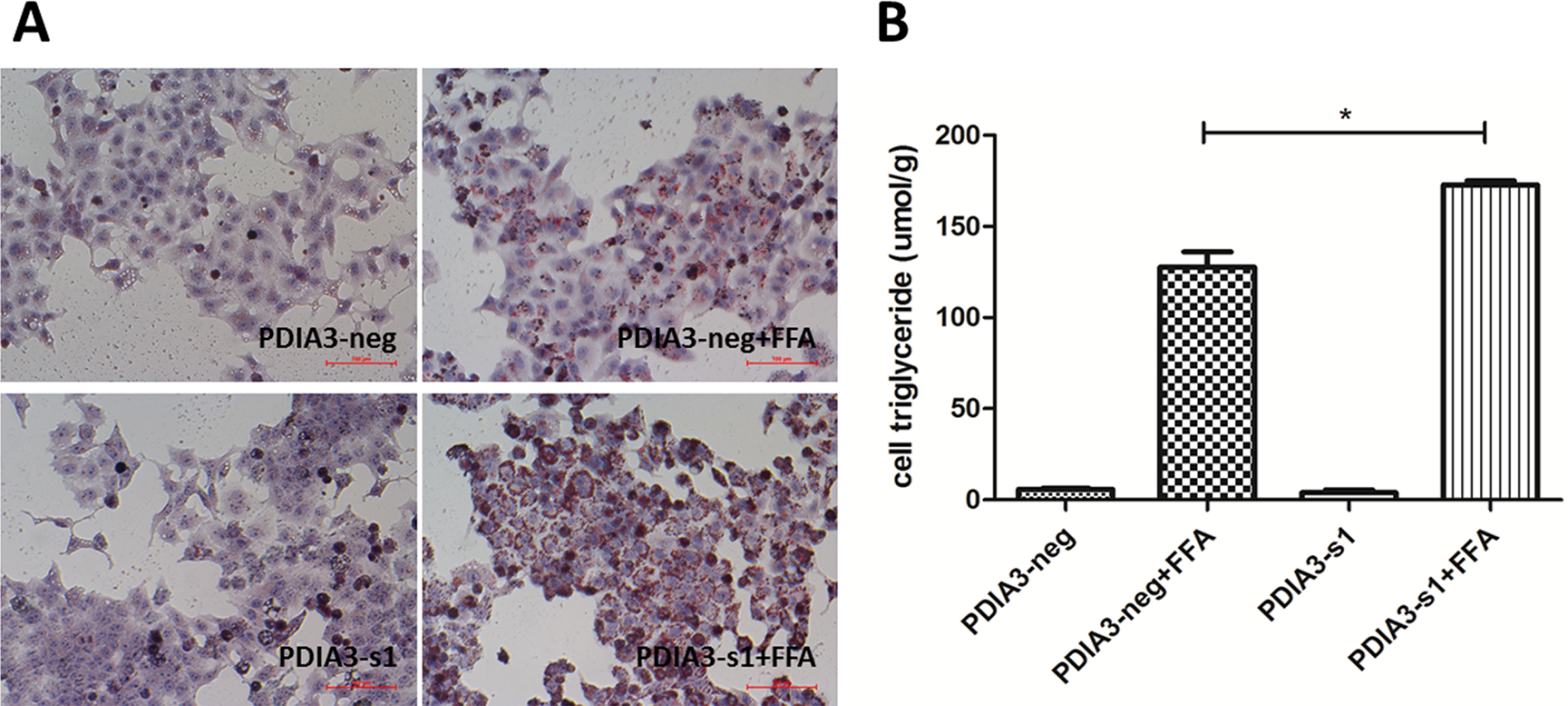
PDIA3 siRNA exacerbates hepatocellular steatosis in L02 cells. (A) Oil red O staining of cells treats with or without FFA after transfection of PDIA3-s1 or PDIA3-neg. Original magnification, 200×. (B) Influence of PDIA3 knockdown on intracellular triglyceride level induced by FFA. These experiments were repeated three times. *P<0.05.

To explore the role of PDIA3 knockdown on lipid accumulation, L02 cells transfected with PDIA3 siRNA (PDIA3-s1) or negative siRNA (PDIA3-neg) were cultured in full media with or without FFA. Oil red O staining demonstrated that increased lipid stores were present in hepatocytes when cells were treated with FFA for 24 hours (Fig 3A). The increases were more obvious in cells transfected with PDIA3 siRNA in FFA medium, however, in FFA free medium, transfection of PDIA3 siRNA seemed no influence on lipid stores. Meanwhile, the triglyceride assay showed the accordingly results (Fig 3B; *P*<0.05).

### PDIA3 siRNA aggravated cellular apoptosis of steatotic L02 cells

**Fig 4:**
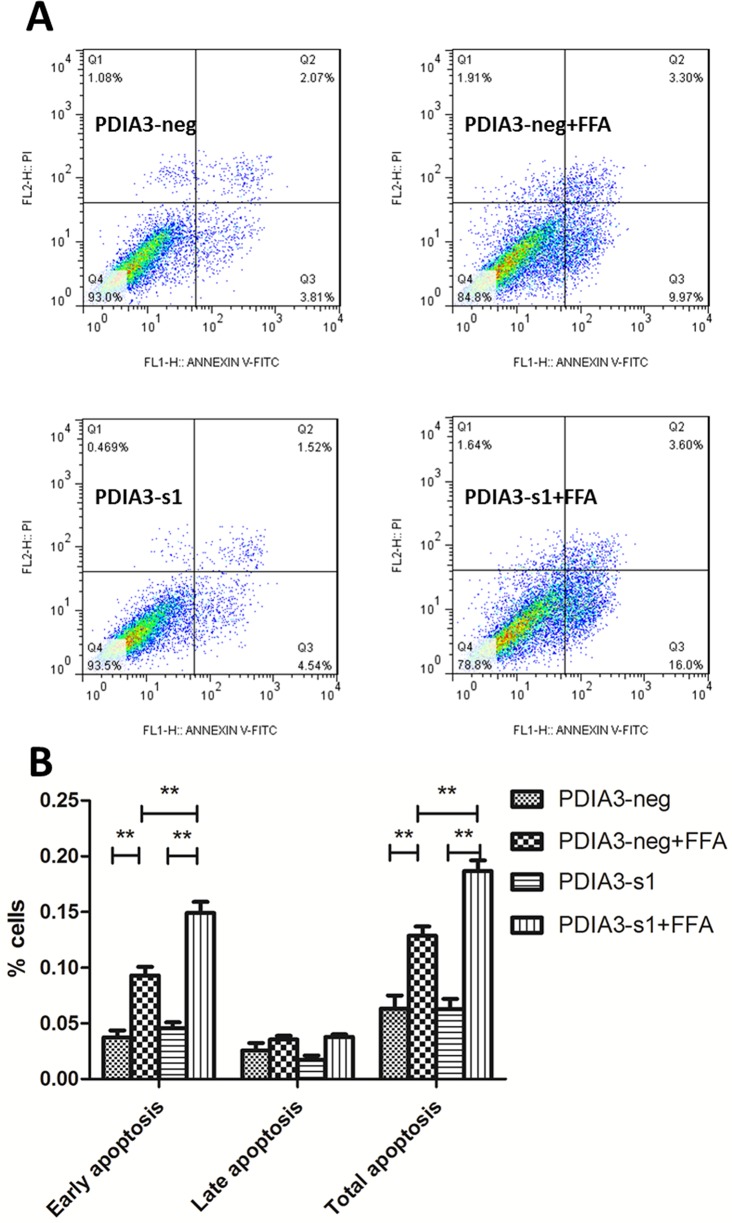
PDIA3 siRNA aggravates cellular apoptosis of steatotic L02 cells. (A) Flow cytometry scatter plot after Annexin V-FITC/PI staining: left lower quadrant: normal cells; right lower quadrant: early apoptosis cells (FITC Annexin V positive and PI negative); right upper quadrant: late apoptosis cells (both FITC Annexin V and PI positive); left upper quadrant: necrotic cells. The percentage represents the proportion of cells in each quadrant. (B) The histogram of early, late and total apoptosis after three independent experiments. **P<0.01.

We next examined whether PDIA3 was involved in FFA-induced cellular apoptosis. Flow cytometer detecting cellular apoptosis revealed that early apoptosis was increased when L02 cells were treated with FFA for 24 hours, while there was no influence on late apoptosis. The siRNA knockdown of PDIA3 resulted in a significant increase of early apoptosis in FFA medium. However, in FFA free medium, transfection of PDIA3 had no influence on cellular apoptosis (Fig 4A). Results of three independent experiments suggested that the difference was significant (Fig 4B; *P*<0.01).

### Knockdown of PDIA3 increased the expression level of FAS

**Fig 5:**
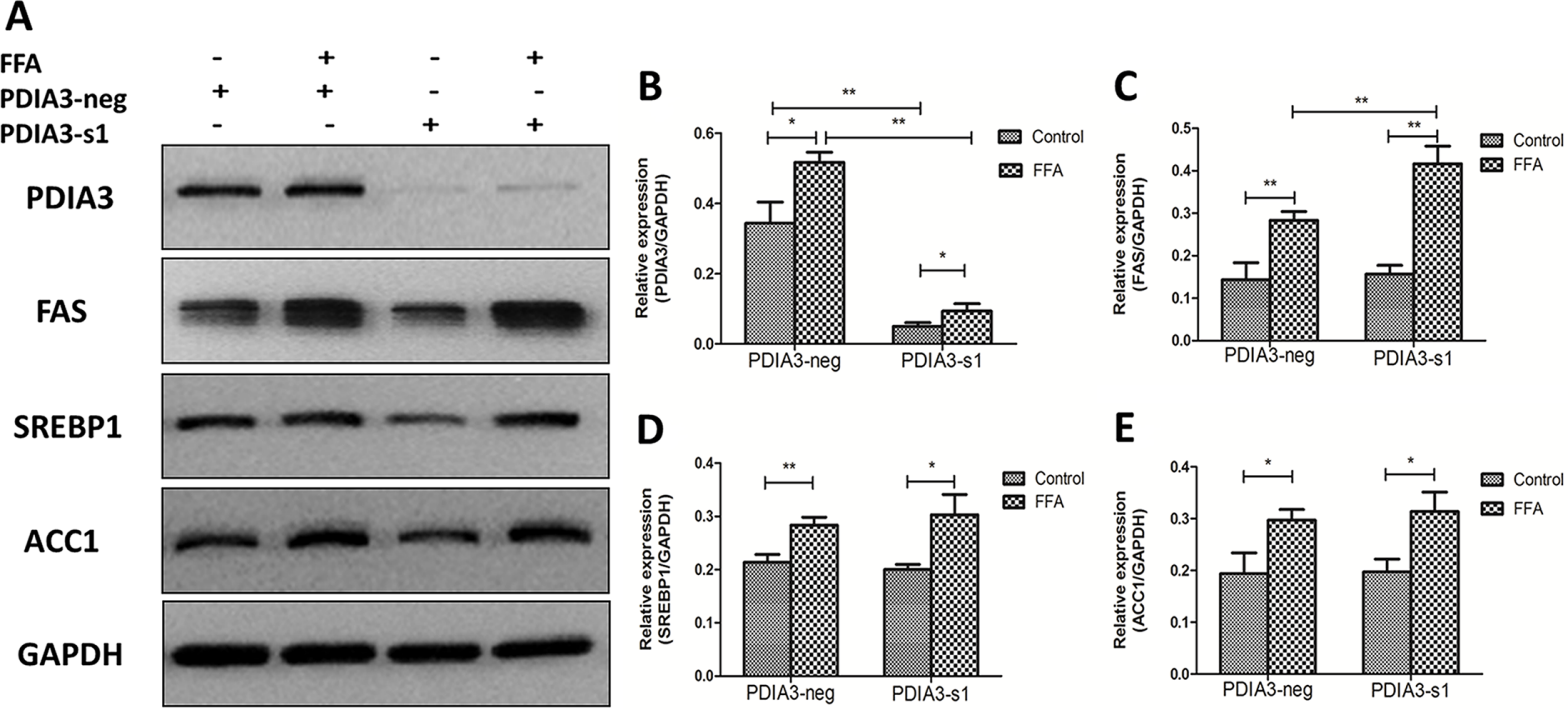
The expression level of lipid synthesis proteins. (A) Western blot analysis of PDIA3, FAS, SREBP1 and ACC1 proteins expression. (B.C.D.E) The relative expression levels quantified by densitometry analysis of bands and normalized to GAPDH protein. These experiments were repeated three times in triplicate. *P<0.05, **P<0.01.

In order to explore the underlying mechanism by which PDIA3 siRNA exacerbated hepatocellular steatosis in L02 cells, Western Blot was used to detect several proteins involved in lipid synthesis: fatty acid synthase (FAS), sterol regulatory element binding protein1 (SREBP1) and Acetyl CoA Carboxylase (ACC1). We found that PDIA3, FAS, SREBP1 and ACC1 were all up-regulated when L02 cells were treated with FFA for 24 hours. siRNA-mediated knockdown of PDIA3 resulted in significant increase in FAS protein expression level as compared with negative siRNA after FFA treatment, while SREBP1 and ACC1 showed no statistically difference. In FFA free medium, PDIA3 knockdown had no significant effect on FAS, SREBP1 and ACC1 expression (Fig 5).

### Knockdown of PDIA3 aggravated FFA-induced cell apoptosis through PERK-CHOP pathway

**Fig 6:**
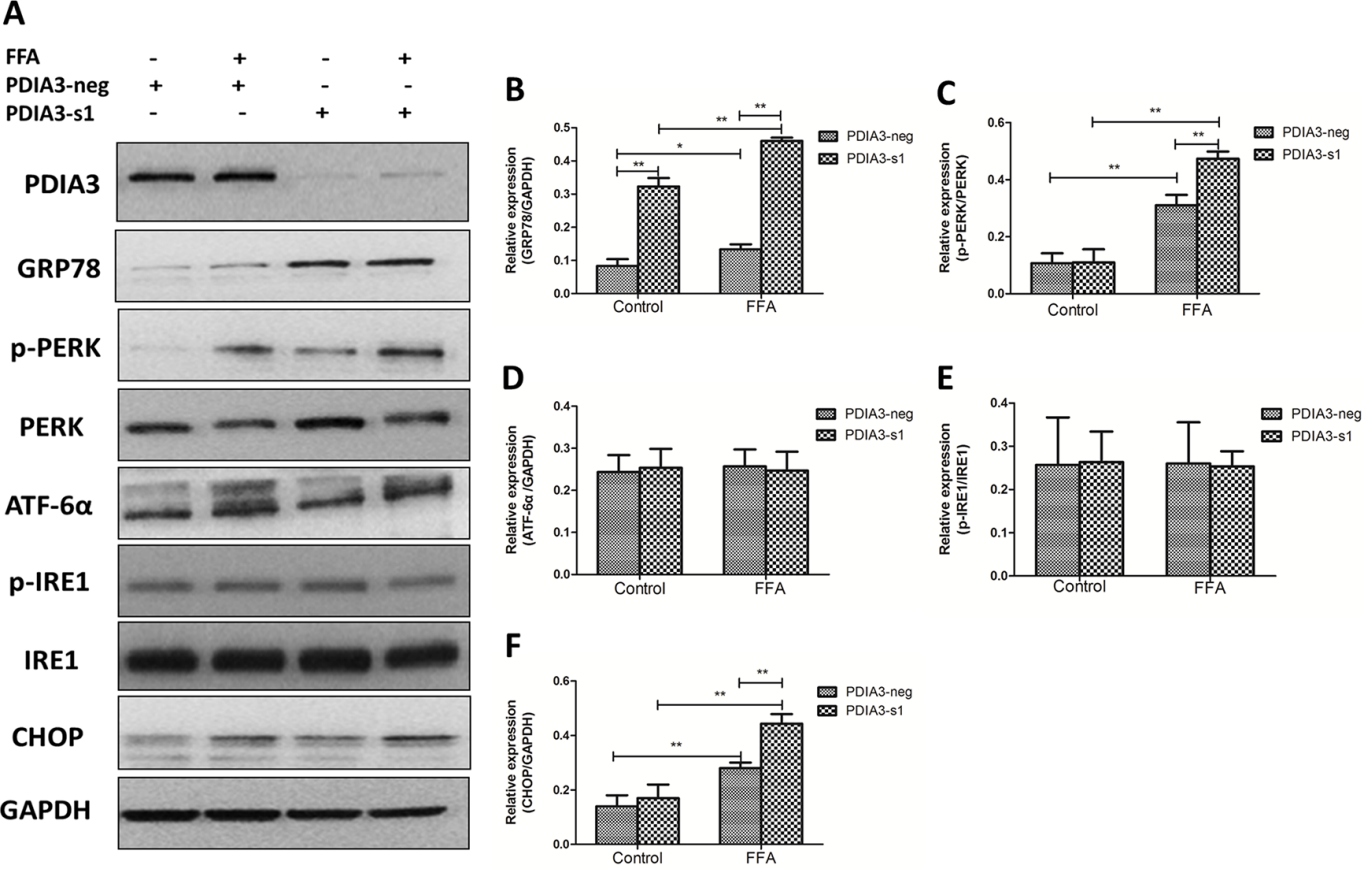
The expression level of ERS and apoptosis related proteins. (A) Western blot analysis of GRP78, PERK, p-PERK, ATF-6α, IRE1, p-IRE1 and CHOP proteins expression. (B.C.D.E.F) The relative expression levels quantified by densitometry analysis of bands and normalized to GAPDH protein. These experiments were repeated three times in triplicate. *P<0.05, **P<0.01.

Since PDIA3 knockdown aggravated hepatocellular apoptosis, and PDIA3 is an important endoplasmic reticulum molecular chaperone, we speculated that PDIA3 might affect cellular apoptosis through ERS pathway. To clarify the potential mechanism, Western Blot was used to detect the ERS related proteins: glucose-regulated protein 78 (GRP78), PERK, phospho-PERK (p-PERK), ATF-6α, IRE1 and phospho-IRE1 (p-IRE1). The expression level of C/EBP homologous protein (CHOP), the most well-characterized mediator of ER stress-induced cell death, was also detected. The results showed that GRP78, p-PERK and CHOP were up-regulated when L02 cells were treated with FFA for 24 hours, while p-IRE1 and ATF-6α had no obvious difference. PDIA3 knockdown resulted in significant increases in GRP78 and p-PERK level as compared with negative siRNA after FFA treatment. PDIA3 knockdown also made a further up-regulation of CHOP level (Fig 6).

## Discussion

NAFLD patients exhibit increased peripheral adipose decomposition and cyclic high FFA [21]. Prolonged high level of cyclic FFA can cause a series of metabolic obstacles, which eventually lead to hepatic steatosis [22]. Therefore, cellular FFA loading is commonly employed to develop *in vitro* models of steatosis. In our research, sodium palmitate was used at the final concentration of 0.5 mM to establish fat loaded cell models of NAFLD, then oil red O staining and cellular triglyceride assay suggested the success. The induction concentration and duration were determined by related references [23,24], selecting a condition to cause some damage to hepatocytes but not so many apoptotic cells.

A putative mechanism linking FFA to NAFLD may been endoplasmic reticulum stress (ERS). A certain concentration of FFA, especially long chain saturated FFA (such as PA) can successfully induce ERS in a variety of cell lines, including hepatocytes [15,16]. In the current study, after treated with sodium palmitate, Western Blot proved that GRP78 and phosphorylated PERK were up-regulated, while the other two sensors, ATF6α and IRE1, remained almost dormant. GRP78 is an intraluminal chaperone, which is bound to the three transmembrane sensors when the endoplasmic reticulum is unstressed. While ERS occurred, GRP78 is displaced from these stress sensors, leading to their activation, so it is regarded as the major biomarker of ERS [25]. PERK and IRE1 undergo dimerization and transautophosphorylation when activation and ATF6α is activated by regulated intramembrane proteolysis in the Golgi [26]. Our results indicate that the PERK signaling pathway plays a key role in mediating sodium palmitate-induced ERS, which is consistent with some previous studies [27,28].

PDIA3 mainly serves as molecular chaperone in the endoplasmic reticulum, which can be induced when secretary protein excessive synthesis, as well as the accumulation of unfolded or misfolded proteins in the endoplasmic reticulum. It was reported that PDIA3 was highly induced in neurodegenerative disease like prion disease, renal fibrosis, obese insulin-resistant nondiabetic individuals and livers of rats fed high-fat diet [18–20,29,30]. However, the potential role of PDIA3 in saturated FFA induced cell models of NAFLD remains to be determined. Our data pointed out that PDIA3 knockdown induced the further up-regulation of GRP78 and p-PERK proteins after FFA treatment, suggesting that PDIA3 knockdown aggravates FFA-induced ERS. However, in FFA free medium, PDIA3 knockdown only induced GRP78 protein up-regulated, the possible explanation may owe to their similar function of endoplasmic reticulum molecular chaperone. PDIA3 knockdown increases the expression of GRP78 compensatory.

Using the oil red O staining and triglyceride assay to investigate the effect of PDIA3 in hepatic steatosis, we found that PDIA3 knockdown may exacerbate FFA-induced cellular steatosis accompanied by the up-regulation of FAS protein expression, while the effect was not observed in FFA free medium. FAS is a key enzyme of organisms endogenous fatty acid synthesis, it catalyzes acetyl CoA and malonyl CoA to form long-chain fatty acid [31]. Some scholars put forward that PDIA3 can interact with nuclear DNA to influence the expression of certain genes [32]. What’s more, deletion of PERK has been reported to inhibit the sustained expression of FAS [27]. So we speculated that PDIA3 knockdown influences the lipid deposition of steatotic hepatocytes by regulating the expression of FAS directly or indirectly. However, the mechanisms that how PDIA3 affects lipid metabolism in this hepatocyte cell models need further investigation. How does PDIA3 regulate FAS expression? Do they have any direct or indirect interactions? How does PDIA3 affect the post-translation of lipogenesis enzymes and AMPK? These questions will be further studied in our next study.

Finally, our study also detected that PDIA3 knockdown promoted steatotic heptocytes apoptosis along with the up-regulation of CHOP protein. Increasing evidence suggests that prolonged, severe ERS can target the cell for apoptosis via a number of putative mechanisms, including extrusion of luminal calcium, activation of CHOP and c-Jun N-terminal kinase (JNK) [33–35]. CHOP is an ER-resident transcription factor that functions downstream of the transmembrane protein PERK and ATF6. It is perhaps the most well-characterized mediator of ER stress-induced cell death [28]. In the current study we find that PDIA3 knockdown exacerbate hepatocytes ERS and apoptosis induced by sodium palmitate, and the potential mechanism may be associated with the activation of PERK-CHOP pathway.

**Fig 7:**
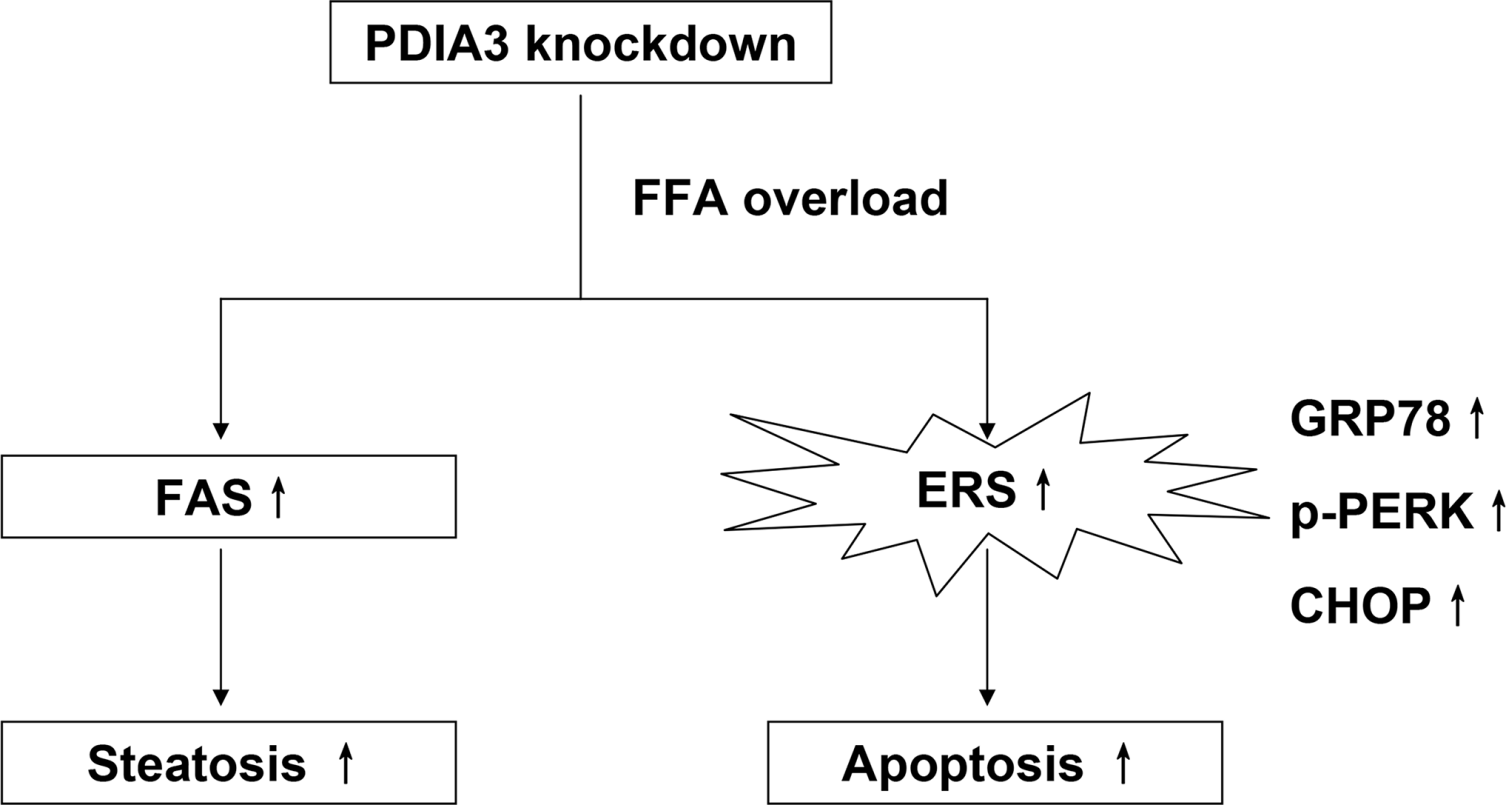
The schematic diagram illustrating the potential role of PDIA3 in the FFA-induced hepatocyte steatosis and apoptosis in L02 cells.

Among the results in present study, PDIA3 knockdown seems only affect ERS level, lipid synthesis, degree of apoptosis and their related protein expression in FFA medium, and it may owe to that FFA condition changes the control system of hepatocytes. To sum up, our study reveals the function of PDIA3 in the NAFLD cell models, and puts forward the possible molecular mechanism (Fig 7). PDIA3 is promising to be the molecular diagnosis and treatment target of NAFLD.

## References

[1] Bedogni G, Miglioli L, Masutti F, Tiribelli C, Marchesini G, Bellentani S. (2005) Prevalence of and risk factors for nonalcoholic fatty liver disease: the Dionysos nutrition and liver study. Hepatology 42(1): 44–52. 15895401 10.1002/hep.20734

[2] Fan JG, Farrell GC. (2009) Epidemiology of non-alcoholic fatty liver disease in China. J Hepatol 50(1): 204–210. 10.1016/j.jhep.2008.10.010 19014878

[3] Farrell GC, Larter CZ. (2006) Nonalcoholic fatty liver disease: from steatosis to cirrhosis. Hepatology 43(2 Suppl 1): S99–S112. 16447287 10.1002/hep.20973

[4] Bugianesi E, Leone N, Vanni E, Marchesini G, Brunello F, Carucci P, et al (2002) Expanding the natural history of nonalcoholic steatohepatitis: from cryptogenic cirrhosis to hepatocellular carcinoma. Gastroenterology 123(1): 134–140. 12105842 10.1053/gast.2002.34168

[5] Day CP, James OF. (1998) Steatohepatitis: a tale of two "hits"? Gastroenterology 114(4): 842–845. 9547102 10.1016/s0016-5085(98)70599-2

[6] Puri P, Mirshahi F, Cheung O, Natarajan R, Maher JW, Kellum JM, et al (2008) Activation and dysregulation of the unfolded protein response in nonalcoholic fatty liver disease. Gastroenterology 134(2): 568–576. 18082745 10.1053/j.gastro.2007.10.039

[7] Rinella ME, Siddiqui MS, Gardikiotes K, Gottstein J, Elias M, Green RM. (2011) Dysregulation of the unfolded protein response in db/db mice with diet-induced steatohepatitis. Hepatology 54(5): 1600–1609. 10.1002/hep.24553 21748768 PMC3205284

[8] Xu C, Bailly-Maitre B, Reed JC. (2005) Endoplasmic reticulum stress: cell life and death decisions. J Clin Invest 115(10): 2656–2664. 16200199 10.1172/JCI26373 PMC1236697

[9] Bravo R, Parra V, Gatica D, Rodriguez AE, Torrealba N, Paredes F, et al (2013) Endoplasmic reticulum and the unfolded protein response: dynamics and metabolic integration. Int Rev Cell Mol Biol 301: 215–290. 10.1016/B978-0-12-407704-1.00005-1 23317820 PMC3666557

[10] Diehl JA, Fuchs SY, Koumenis C. (2011) The cell biology of the unfolded protein response. Gastroenterology 141(1): 38–41.e2. 10.1053/j.gastro.2011.05.018 21620842 PMC3129462

[11] Walter P, Ron D. (2011) The unfolded protein response: from stress pathway to homeostatic regulation. Science; 334(6059): 1081–1086. 10.1126/science.1209038 22116877

[12] Kim I, Xu W, Reed JC (2008) Cell death and endoplasmic reticulum stress: disease relevance and therapeutic opportunities. Nat Rev Drug Discov 7(12): 1013–1030. 10.1038/nrd2755 19043451

[13] Hotamisligil GS. (2010) Endoplasmic reticulum stress and the inflammatory basis of metabolic disease. Cell 140(6): 900–917. 10.1016/j.cell.2010.02.034 20303879 PMC2887297

[14] Glimcher LH, Lee AH. (2009) From sugar to fat: How the transcription factor XBP1 regulates hepatic lipogenesis. Ann N Y Acad Sci 1173 Suppl 1: E2–E9. 10.1111/j.1749-6632.2009.04956.x 19751410 PMC3096021

[15] Pagliassotti MJ, Wei Y, Wang D. (2007) Insulin protects liver cells from saturated fatty acid-induced apoptosis via inhibition of c-Jun NH2 terminal kinase activity. Endocrinology 148(7): 3338–3345. 17431009 10.1210/en.2006-1710

[16] Wei Y, Wang D, Topczewski F, Pagliassotti MJ. (2006) Saturated fatty acids induce endoplasmic reticulum stress and apoptosis independently of ceramide in liver cells. Am J Physiol Endocrinol Metab 291(2): E275–E281. 16492686 10.1152/ajpendo.00644.2005

[17] Turano C1, Gaucci E, Grillo C, Chichiarelli S. (2011) ERp57/GRP58: a protein with multiple functions. Cell Mol Biol Lett 16(4):539–563. 10.2478/s11658-011-0022-z 21837552 PMC6275603

[18] Hetz C, Russelakis-Carneiro M, Walchli S, Carboni S, Vial-Knecht E, Maundrell K, et al (2005) The disulfide isomerase Grp58 is a protective factor against prion neurotoxicity. J Neurosci 25(11):2793–2802. 15772339 10.1523/JNEUROSCI.4090-04.2005 PMC6725139

[19] Hetz CA, Soto C. (2006) Stressing out the ER: a role of the unfolded protein response in prion-related disorders. Curr Mol Med 6(1):37–43. 16472111 10.2174/156652406775574578 PMC2838391

[20] Zhang X, Yang J, Guo Y, Ye H, Yu C, Xu C, et al (2010) Functional proteomic analysis of nonalcoholic fatty liver disease in rat models: enoyl-coenzyme a hydratase down-regulation exacerbates hepatic steatosis. Hepatology 51(4): 1190–1199. 10.1002/hep.23486 20162621

[21] Marra F, Gastaldelli A, Svegliati BG, Tell G, Tiribelli C (2008) Molecular basis and mechanisms of progression of non-alcoholic steatohepatitis. Trends Mol Med 14: 72–81. 10.1016/j.molmed.2007.12.003 18218340

[22] Parekh S, Anania FA (2007) Abnormal lipid and glucose metabolism in obesity: implications for nonalcoholic fatty liver disease. Gastroenterology 132: 2191–2207. 17498512 10.1053/j.gastro.2007.03.055

[23] Cazanave SC, Elmi NA, Akazawa Y, Bronk SF, Mott JL, Gores GJ. (2010) CHOP and AP-1 cooperatively mediate PUMA expression during lipoapoptosis. Am J Physiol Gastrointest Liver Physiol 299: G236–G243. 10.1152/ajpgi.00091.2010 20430872 PMC2904106

[24] Cao J, Feng XX, Yao L, Ning B, Yang ZX, Fang DL, et al (2014) Saturated free fatty acid sodium palmitate-induced lipoapoptosis by targeting glycogen synthase kinase-3beta activation in human liver cells. Dig Dis Sci 59: 346–357. 10.1007/s10620-013-2896-2 24132507

[25] Rutkowski DT, Kaufman RJ (2007) That which does not kill me makes me stronger: adapting to chronic ER stress. Trends Biochem Sci 32: 469–476. 17920280 10.1016/j.tibs.2007.09.003

[26] Gentile CL, Frye M, Pagliassotti MJ (2011) Endoplasmic reticulum stress and the unfolded protein response in nonalcoholic fatty liver disease. Antioxid Redox Signal 15: 505–521. 10.1089/ars.2010.3790 21128705 PMC3118611

[27] Bobrovnikova-Marjon E, Hatzivassiliou G, Grigoriadou C, Romero M, Cavener DR, Thompson CB, et al (2008) PERK-dependent regulation of lipogenesis during mouse mammary gland development and adipocyte differentiation. Proc Natl Acad Sci U S A 105: 16314–16319. 10.1073/pnas.0808517105 18852460 PMC2570995

[28] Cao J, Dai DL, Yao L, Yu HH, Ning B, Zhang Q, et al (2012) Saturated fatty acid induction of endoplasmic reticulum stress and apoptosis in human liver cells via the PERK/ATF4/CHOP signaling pathway. Mol Cell Biochem 364: 115–129. 10.1007/s11010-011-1211-9 22246806

[29] Dihazi H, Dihazi GH, Bibi A, Eltoweissy M, Mueller CA, Asif AR, et al (2013) Secretion of ERP57 is important for extracellular matrix accumulation and progression of renal fibrosis, and is an early sign of disease onset. J Cell Sci 126(16): 3649–3663. 23781031 10.1242/jcs.125088

[30] Boden G, Merali S (2011) Measurement of the increase in endoplasmic reticulum stress-related proteins and genes in adipose tissue of obese, insulin-resistant individuals. Methods Enzymol 489: 67–82. 10.1016/B978-0-12-385116-1.00004-2 21266224 PMC3249655

[31] Smith S, Witkowski A, Joshi AK (2003) Structural and functional organization of the animal fatty acid synthase. Prog Lipid Res 42(4): 289–317. 12689621 10.1016/s0163-7827(02)00067-x

[32] Coe H, Michalak M (2010) ERp57, a multifunctional endoplasmic reticulum resident oxidoreductase. Int J Biochem Cell Biol 42(6): 796–799. 10.1016/j.biocel.2010.01.009 20079872

[33] Malhi H, Kaufman RJ (2011) Endoplasmic reticulum stress in liver disease. J Hepatol 54: 795–809. 10.1016/j.jhep.2010.11.005 21145844 PMC3375108

[34] Urano F, Wang X, Bertolotti A, Zhang Y, Chung P, Harding HP, et al (2000) Coupling of stress in the ER to activation of JNK protein kinases by transmembrane protein kinase IRE1. Science 287(5453): 664–666. 10650002 10.1126/science.287.5453.664

[35] Oyadomari S, Mori M (2004) Roles of CHOP/GADD153 in endoplasmic reticulum stress. Cell Death Differ 11(4): 381–389. 14685163 10.1038/sj.cdd.4401373

